# Supplementary material for: Enhanced Photovoltaic Properties of Perovskite Solar Cells by Employing Bathocuproine/Hydrophobic Polymer Films as Hole-Blocking/Electron-Transporting Interfacial Layers
Source: Polymers (Basel). 2020 Dec 24;13(1):42. doi: 10.3390/polym13010042 (PMC7795380; doi:10.3390/polym13010042)
Supplement: Supplementary file 1 [file polymers-13-00042-s001.pdf]

polymers

MDPI

Article

Enhanced Photovoltaic Properties of Perovskite Solar Cells by Employing Bathocuproine/Hydrophobic Polymer Films as Hole-Blocking/Electron-Transporting Interfacial Layers

Guan-Zhi Liu [1], Chi-Shiuan Du [1], Jeng-Yue Wu [1], Bo-Tau Liu [2,*], Tzong-Ming Wu [3], Chih-Feng Huang [1] and Rong-Ho Lee [1,*]

[1] Department of Chemical Engineering, National Chung Hsing University, Taichung 402, Taiwan; as798320@gmail.com (G.-Z.L.); justduit0831@gmail.com (C.-S.D.); s0916871303@gmail.com (J.-Y.W.); HuangCF@dragon.nchu.edu.tw (C.-F.H.)

[2] Department of Chemical and Materials Engineering, National Yunlin University of Science and Technology, Yunlin 64002, Taiwan

[3] Department of Materials Science and Engineering, National Chung Hsing University, Taichung 402, Taiwan; tmwu@nchu.edu.tw

* Correspondence: liubo@yuntech.edu.tw (B.-T.L.); rhl@dragon.nchu.edu.tw (R.-H.L.); Tel.: +886-4-22854308 (B.-T.L.); Tel.: +886-4-22854308 (R.-H.L); Fax: +886-4-22854734 (B.-T.L.); +886-4-22854734 (R.-H.L)

Citation: Liu, G.-Z.; Du, C.-S.; Wu, J.-Y.; Liu, B.-T.; Wu, T.-M.; Huang, C.-F.; Lee, R.-H. Enhanced Photovoltaic Properties of Perovskite Solar Cells by Employing Bathocuproine/Hydrophobic Polymer Films as Hole-Blocking/Electron-Transporting Interfacial Layers. *Polymers* **2020**, *13*, 42. https://doi.org/10.3390/Polym13010042

Received: 26 November 2020
Accepted: 22 December 2020
Published: 24 December 2020

Publisher's Note: MDPI stays neutral with regard to jurisdictional claims in published maps and institutional affiliations.

Copyright: © 2020 by the authors. Licensee MDPI, Basel, Switzerland. This article is an open access article distributed under the terms and conditions of the Creative Commons Attribution (CC BY) license (http://creativecommons.org/licenses/by/4.0/).

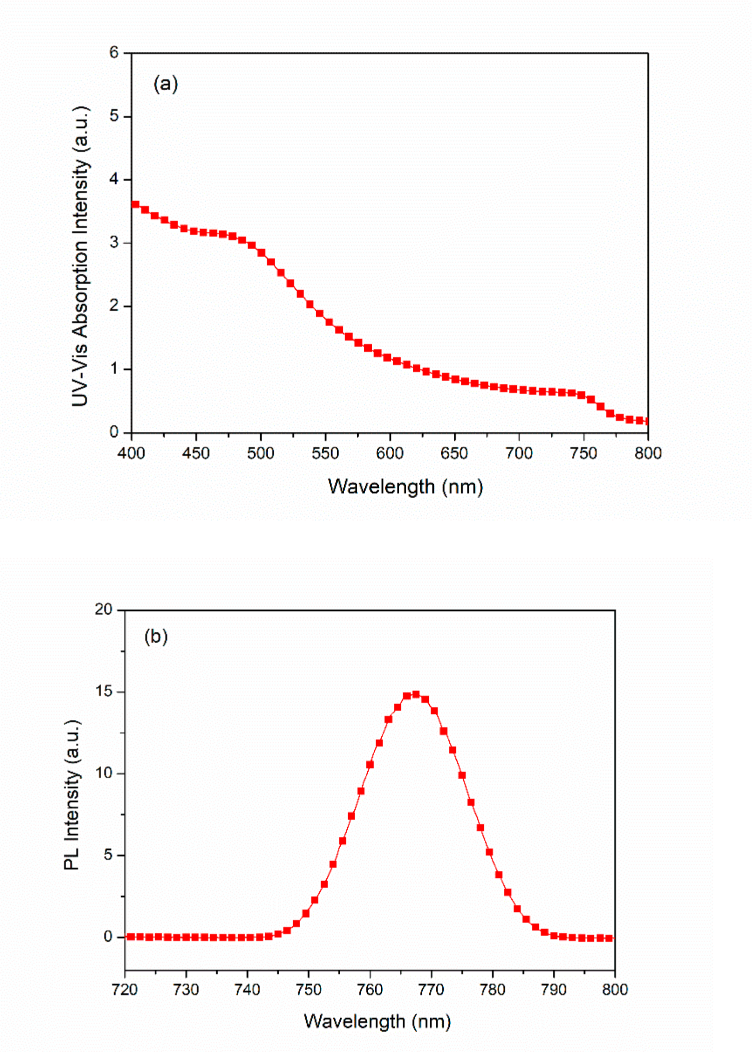

Figure S1. (**a**) UV–Vis absorption and (**b**) PL spectra of the $MAPbI_3$ perovskite film.

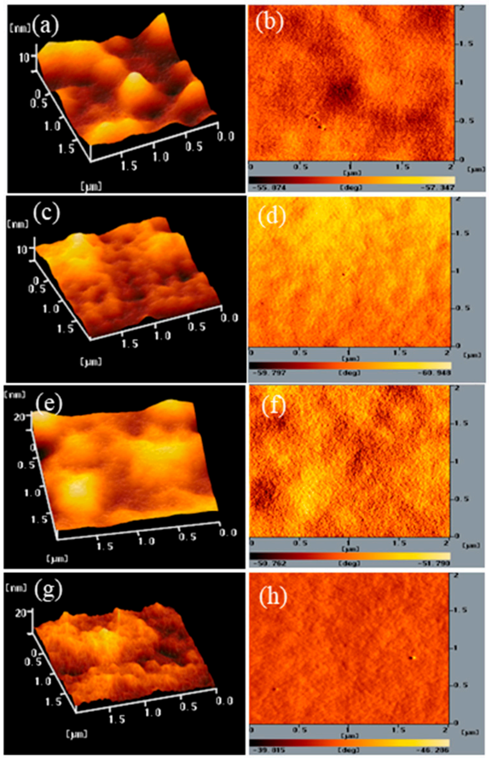

Figure S2. (**a**, **c**, **e**, **g**) Topographic and (**b**, **d**, **f**, **h**) phase AFM images of (**a**, **b**) BCP, (**c**, **d**) BCP/PMMA (10:1, *w*/*w*), (**e**, **f**) BCP/PMMA (5:1, *w*/*w*), and (**g**, **h**) BCP/PMMA (5:2, *w*/*w*) hole-blocking/electron-transporting interfacial layers, recorded after thermal treatment (80 °C, 5 min).

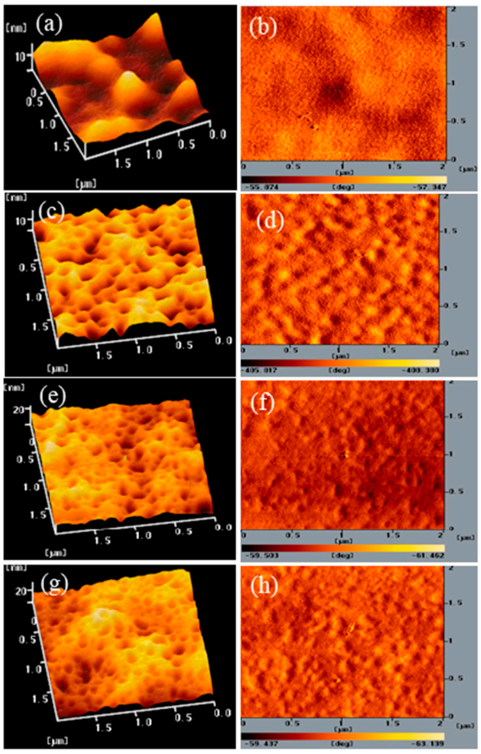

Figure S3. (**a**, **c**, **e**, **g**) Topographic and (**b**, **d**, **f**, **h**) phase AFM images of (**a**, **b**) BCP, (**c**, **d**) BCP/PVP (10:1, *w*/*w*), (**e**, **f**) BCP/PVP (5:1, *w*/*w*), and (**g**, **h**) BCP/PVP (5:2, *w*/*w*) hole-blocking/electron-transporting interfacial layers, recorded after thermal treatment (80 °C, 5 min).

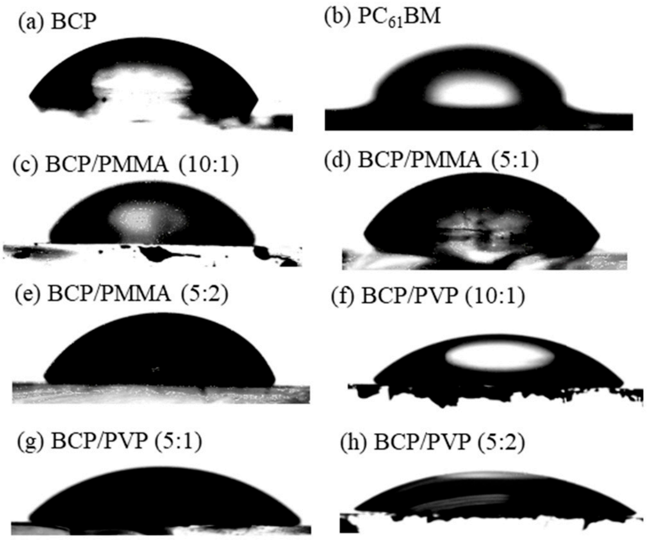

Figure S4. Photographs of water droplets on (**a**) BCP, (**b**) $PC_{61}BM$, (**c**, **d**, **e**) BCP/PMMA (10:1, 5:1, and 5:2, *w*/*w*), and (**f**, **g**, **h**) BCP/PVP (10:1, 5:1, and 5:2, *w*/*w*) films.

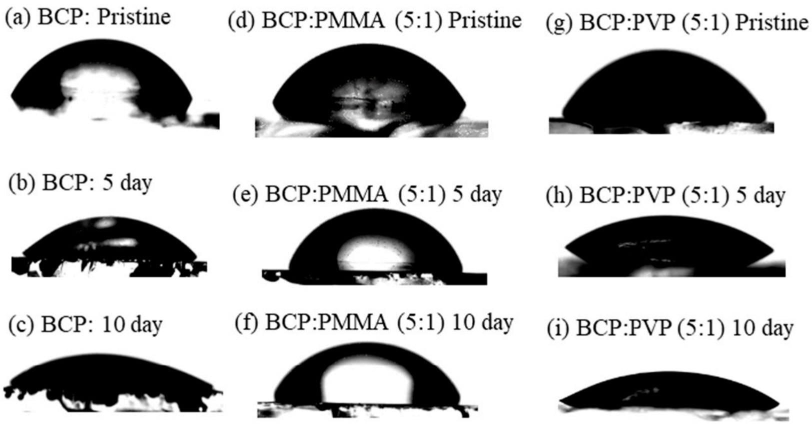

Figure S5. Photographs of water droplets on (**a**–**c**) BCP, (**d**–**f**) BCP/PMMA (5:1, *w*/*w*), and (**g**–**i**) BCP/PVP (5:1, *w*/*w*) films after storage at 30 °C and 35% relative humidity for 0, 5, and 10 days.

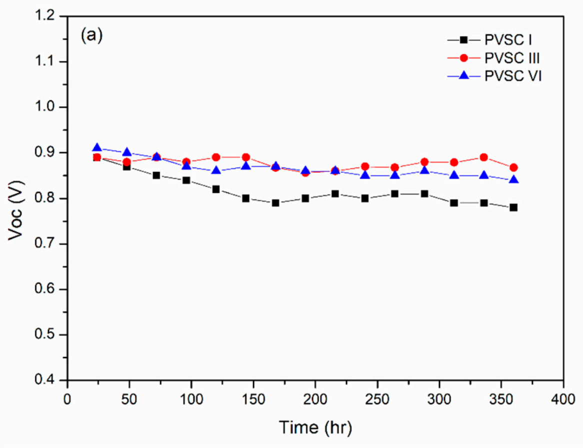

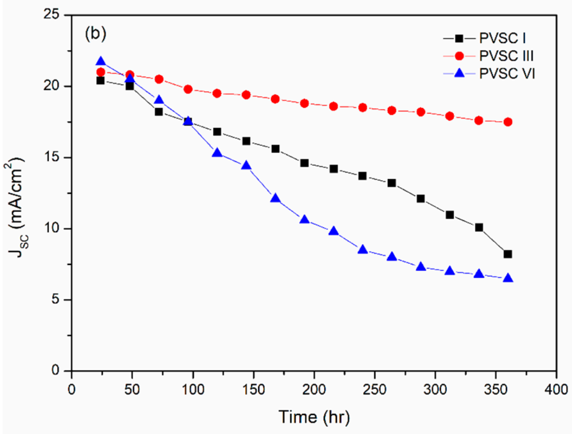

Figure S6. Time dependence of V_{OC} and J_{SC} of the PVSC I, PVSC III, and PVSC VI (measured at 30 °C and 35% relative humidity).

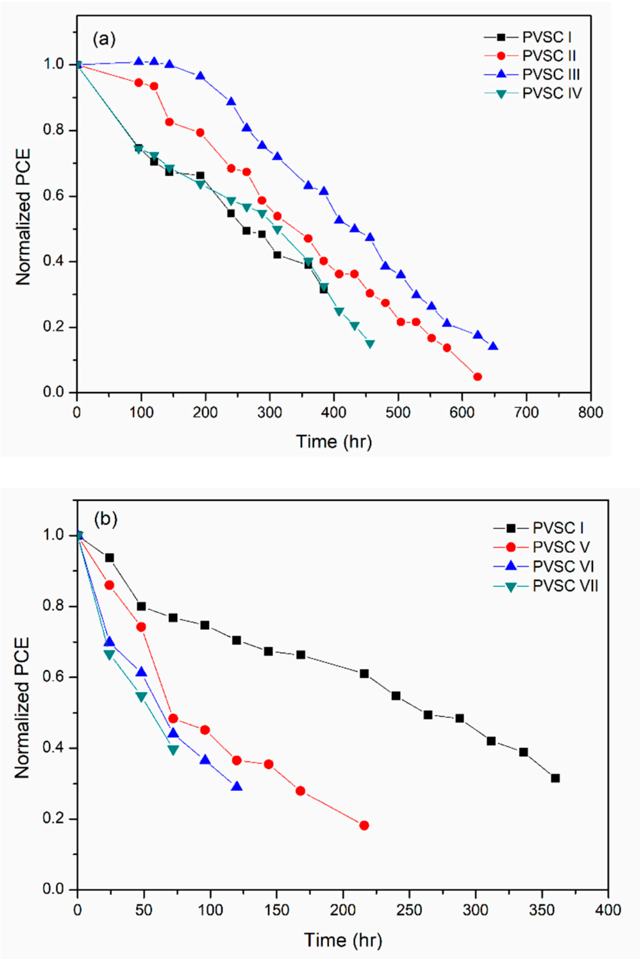

Figure S7. Storage-stability of PVSCs incorporating BCP, BCP/PMMA, and BCP/PVP (measured at 30 °C and 60% relative humidity).

Table S1. Crystal sizes of $MAPbI_3$ film coated with BCP, BCP/PMMA, and BCP/PVP layers after storage at 30 °C and 35% relative humidity for 0, 5, and 10 days.

Sample	Interfacial layer	Time (days)	Crystal size (nm)
I-1	BCP	0	34.21
I-2	BCP	5	29.05
I-3	BCP	10	28.25

II	BCP/PMMA (10:1)	0	-----
III-1	BCP/PMMA (5:1)	0	30.65
III-2	BCP/PMMA (5:1)	5	27.78
III-3	BCP/PMMA (5:1)	10	26.94
IV	BCP/PMMA (5:2)	0	-----
V	BCP/PVP (10:1)	0	-----
VI-1	BCP/PVP (5:1)	0	32.93
VI-2	BCP/PVP (5:1)	5	28.68
VI-3	BCP/PVP (5:1)	10	27.78
VII	BCP/PVP (5:2)	0	----

Table S2. Surface roughnesses and CAs of films of BCP, BCP/PMMA, and BCP/PVP layers after storage at 30 °C and 35% relative humidity for 0, 5, and 10 days.

Sample	Composition (*w/w*)	Time (days)	RMS (nm)	CA (°)
I-1	BCP	0	3.27	74.5
I-2	BCP	5	2.76	49.0
I-3	BCP	10	7.55	37.8
II	BCP/PMMA (10:1)	0	3.25	75.5
III-1	BCP/PMMA (5:1)	0	3.18	78.4
III-2	BCP/PMMA (5:1)	5	5.14	74.4
III-3	BCP/PMMA (5:1)	10	5.31	72.3
IV	BCP/PMMA (5:2)	0	3.29	81.8
V	BCP/PVP (10:1)	0	3.12	58.4
VI-1	BCP/PVP (5:1)	0	3.09	53.5
VI-2	BCP/PVP (5:1)	5	26.45	43.9
VI-3	BCP/PVP (5:1)	10	38.32	35.7
VII	BCP/PVP (5:2)	0	3.15	43.0

Table S3. PV performances of previously reported PVSCs, compared with those measured in this present study.

Device structure	PV performance	Reference
ITO/PEDOT/$MAPbI_3$/$PC_{61}BM$/BCP:PVP/Ag	V_{OC}: 0.92 V; J_{SC}: 21.72 mA cm^{-2}; FF: 0.62; PCE: 12.41%.	This study
ITO/PEDOT/$MAPbI_3$:CDHC/$PC_{61}BM$/Ag	V_{OC}: 0.96 V; J_{SC}: 17.73 mA cm^{-2}; FF: 0.61; PCE: 10.38%.	Cellulose, 2019, 26, 9229-9239.
ITO/PEDOT/$MAPbI_3$/$PC_{61}BM$/Al	V_{OC}: 0.78 V; J_{SC}: 13.2 mA cm^{-2}; FF: 0.60; PCE: 6.2%.	Nanoscale, 2014, 6, 11403–11410.
ITO/PEDOT/$MAPbI_3$:NH_4Cl/$PC_{61}BM$/Al	V_{OC}: 0.88 V; J_{SC}: 14.08 mA cm^{-2}; FF: 0.80; PCE: 9.93%.	Nanoscale, 2014, 6, 9935–9938.
ITO/PEDOT/$MAPbI_3$:PEOXA/$PC_{61}BM$/Al	V_{OC}: 1.04 V; J_{SC}: 8.85 mA cm^{-2}; FF: 0.65; PCE: 6.16%.	RSC Adv., 2015, 5, 775–783.
ITO/PEDOT/$MAPbI_3$/$PC_{61}BM$/Al	V_{OC}: 0.88 V; J_{SC}: 14.16 mA cm^{-2}; FF: 0.60; PCE: 7.6%	Solar Energy Mater. Solar Cells, 2016, 155, 166–175.
ITO/PEDOT/$MAPbI_3$/$PC_{61}BM$/Al	V_{OC}: 0.87 V; J_{SC}: 11.4 mA cm^{-2}; FF: 0.78; PCE: 7.79%.	ACS Appl. Mater. Interfaces, 2017, 9, 32957–32964.
ITO/PEDOT/$MAPbI_3$/$PC_{61}BM$/Ag	V_{OC}: 0.75 V; J_{SC}: 13.76 mA cm^{-2}; FF: 0.40; PCE: 4.13%.	J. Mater. Chem. A, 2017, 5, 12811–12821.

ITO/PEDOT/$MAPbI_3$/$PC_{61}BM$/Ag	V_{OC}: 0.95 V; J_{SC}: 16.55 mA cm^{-2}; FF: 0.59; PCE: 9.29%.	ACS Appl. Mater. Interfaces, 2017, 9, 32957–32964.
